# OUT AND ABOUT? PARTICIPATION IN OUT-OF-HOME ACTIVITIES AND PLACES AMONG PEOPLE WITH DEMENTIA IN MULTIPLE COUNTRIES

**DOI:** 10.1093/geroni/igz038.2846

**Published:** 2019-11-08

**Authors:** Habib Chaudhury, Louise Nygard, Malcolm Cutchin

**Affiliations:** 1 Simon Fraser University, Vancouver, British Columbia, Canada; 2 Karolinska Institute, Stockholm, Sweden; 3 Wayne State University, Detroit, Michigan, United States

## Abstract

Maintaining participation in activities and places in the community can be challenging for people with dementia in the face of cognitive decline. These challenges could be related to driving cessation, accessing transit, ability to navigate in the community, socially uncomfortable situations and functionally difficult tasks. Consequently, people with dementia are at risk of progressively becoming isolated from the community and being unable to meaningfully age-in-place in the home and community. Familiar and responsive neighborhood built environmental features, public awareness of dementia and appropriate support, dementia-friendly transit systems, are among the potential socio-physical environmental factors to foster maintenance of participation and activities for persons living with dementia. Empirical literature exploring levels of participation outside the home, wayfinding and orientation issues, and the nature and prevalence of challenging circumstances for older adults with dementia in the community is scarce. This session will present four papers in this area based on empirical projects conducted in Sweden, Switzerland, U.K. and Canada. Three of these projects used the recently developed Participation in ACTivities and Places OUTside the Home (ACT-OUT) questionnaire to generate evidence on the levels of participation and perceptions of risks outside the home for people with and without dementia. A presentation will also summarize the theoretical bases for the ACT-OUT research discussed in the other presentations. One study will focus on the influence of Everyday Technology on participation patterns. Implications of the research findings to advance theoretical understanding, generate evidence-base and impact policy and practice, along with future research directions will be considered.

